# Novel elemental grading system for radiographic lumbar spondylosis in a population based-cohort study of a Japanese mountain village

**DOI:** 10.1371/journal.pone.0270282

**Published:** 2022-06-28

**Authors:** Junichi Yamada, Koji Akeda, Norihiko Takegami, Tatsuhiko Fujiwara, Akinobu Nishimura, Akihiro Sudo

**Affiliations:** Department of Orthopaedic Surgery, Mie University Graduate School of Medicine, Tsu, Mie, Japan; Assiut University Faculty of Medicine, EGYPT

## Abstract

**Purpose:**

Lumbar radiography is a primary screening tool for lumbar spondylosis (LS). Kellgren-Lawrence (KL) classification is widely used to evaluate LS; however, it cannot individually evaluate each radiographic feature. The purpose of this study was to 1) evaluate radiographic LS using a novel elemental grading system and 2) investigate the relationship between the grades of radiographic LS and low back pain (LBP) in a population-based cohort study.

**Methods:**

A total of 260 (75 men, 185 women; mean age, 71.5 ± 8.7 years) participants were included in this study. Participants were divided into two groups according to the presence of LBP (LBP- and LBP+ groups). Radiographic features, including osteophyte (OP), disc height narrowing (DHN), vertebral sclerosis (VS), and spondylolisthesis (SL), were classified between grades of 0–2 grades according to the extent of radiographic changes. The sum of grades at each intervertebral level was designated as the intervertebral grade (IG).

**Results:**

Intra- and inter-observer reliability (kappa coefficient) of OP, DHN, VS, and SL were 0.82–0.92. OP, DHN, VS, and IG grades were significantly higher in the LBP+ group than in the LBP- group. There were no significant differences in KL grades between the LBP- and LBP+ groups. Logistic regression analysis demonstrated that VS grade was a significant independent factor associated with LBP.

**Conclusion:**

The novel elemental grading system of LS would reflect LBP more accurately than the KL classification by individually evaluating each radiographic feature.

## Introduction

Low back pain (LBP) is one of the commonest health problems in the elderly, with a lifetime prevalence reported to be as high as 84% [1]. Lumbar radiography is a commonly used screening tool for patients with LBP due to its relatively low cost and ease of administration [2]. Lumbar spondylosis (LS) is more prevalent in the aging population and is reported to be related to low back pain (LBP) [3]. The radiographic features of LS include osteophytes (OP), disc height narrowing (DHN), vertebral sclerosis (VS), and spondylolisthesis (SL). In a systemic review, Raastad et al. reported that radiographic features of LS such as DHN and SL were significantly associated with LBP among occupational and community-based populations [3]; however, OP and VS had a weak or non-significant association with LBP [3]. They also pointed out various epidemiological studies reporting a variety of categorization grades of radiographic features. The Kellgren Lawrence (KL) classification is a semi-quantitative method for comprehensively evaluating radiographic LS, which is characterized by OP, DHN, and VS, and is widely used in epidemiological studies [4–6]. However, it is difficult to evaluate the individual radiographic features of LS separately [7].

Thus, we have established a novel elemental grading system based on the KL classification that can individually grade the radiographic features of LS, including OP, DHN, VS, and SL. Therefore, the purpose of this study was 1) to evaluate LS using a novel elemental grading system of lumbar radiographs and 2) to investigate the relationship between the grade of radiographic LS and LBP in a population-based cohort study.

## Materials and methods

### Participants

Data were analyzed from a population-based longitudinal prospective study of osteoporosis and knee osteoarthritis (OA) in a typical mountain village, Ohdai-cho, in the Mie Prefecture of Japan [8, 9]. This study was conducted with the approval of the Institutional Committee for the Ethics of Human Research, and all participants provided written informed consent before enrollment in the study.

### Clinical interview and physical examination

Participants completed an interviewer-administered questionnaire that included information on age, sex, the presence of chronic LBP (cLBP), LBP intensity, and health-related quality of life (QOL), including the EuroQOL 5-Dimension (EQ-5D), EuroQOL Visual Analogue Scale (EQ-VAS), and Oswestry Disability Index (ODI), as previously reported [10]. LBP intensity was measured using an 11-grade pain intensity numerical rating scale (NRS) [11] (S1 File). EQ-5D has two parts: EQ-5D self-classifier [12] and EQ-VAS [13] (S2 File). ODI, graded from 0 to 100, is the most commonly used questionnaire to assess LBP-related QOL, with higher grades indicating worse condition [14] (S3 File). The bone mineral density (BMD) of the forearm was measured using dual-energy X-ray absorptiometry (DCS-600EX, Aloka, Tokyo). The presence of cLBP was determined by asking, “Do you have low back pain lasting more than three months?” Participants were divided into two groups: the LBP- group and LBP+ group.

### Radiographic assessment of lumbar spondylosis using novel elemental grading system

**Fig 1:**
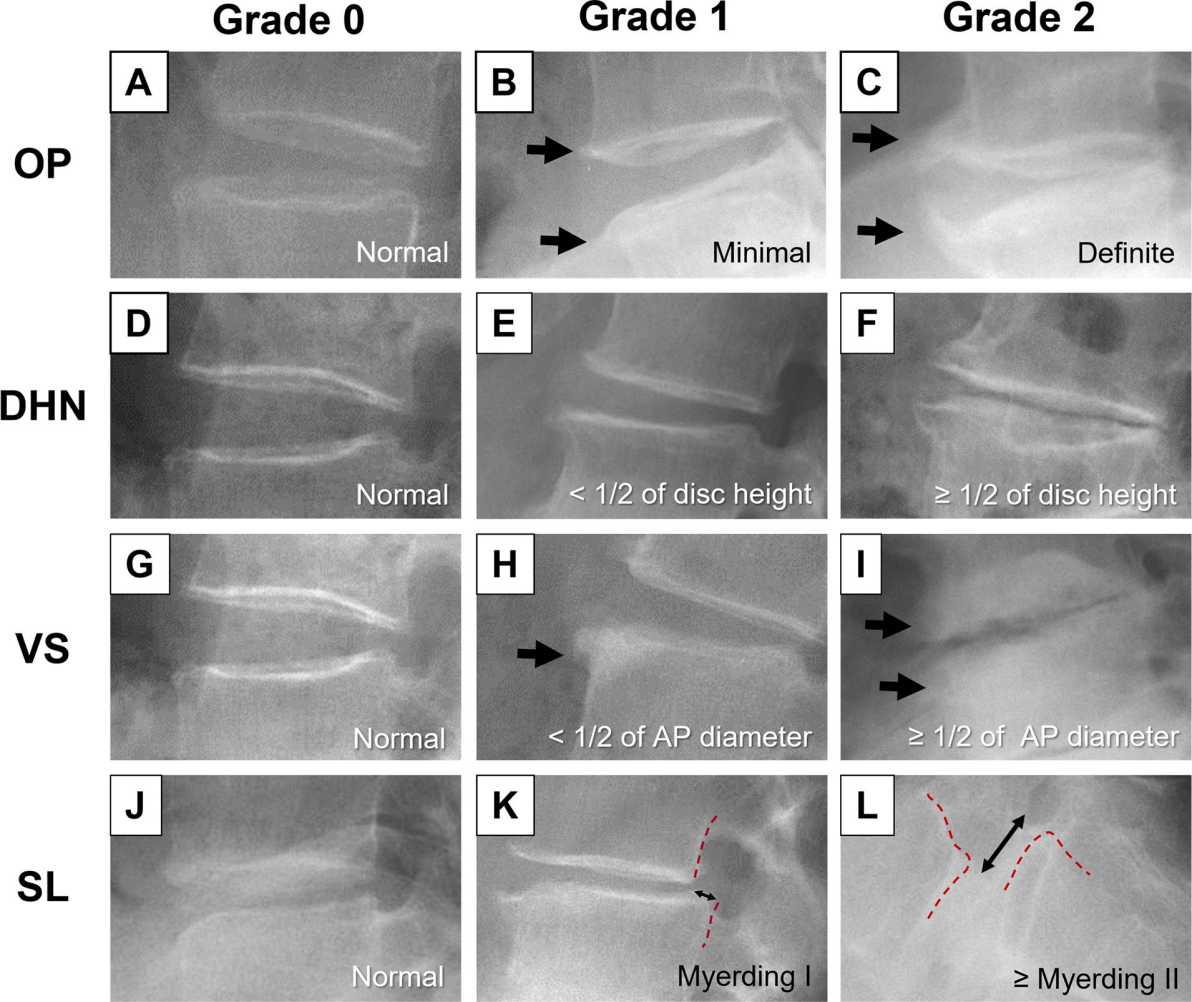
Typical radiographic features for the novel elemental grading system. Osteophytes (OP) were classified as grade 0, normal (A); grade 1, minimal anterior vertebral osteophytosis (B); and grade 2, definite vertebral osteophytosis (C). Disc height narrowing (DHN) was classified as grade 0, normal (D); grade 1, mild (less than 1/2 of estimated disc height) DHN (E); and grade 2, severe (equal to or more than 1/2 of estimated disc height) DHN (F). Vertebral sclerosis (VS) was classified into grade 0, normal (G); grade 1, mild (less than 1/2 of anteroposterior diameter) sclerosis (H); and grade 2, severe (equal to or more than 1/2 of anteroposterior diameter) sclerosis (I). Spondylolisthesis (SL) was classified into grade 0, normal (J); grade 1, Meyerding [15] grade I (K); and grade 2, Meyerding grade II or worse (L).

Lateral lumbar spine radiographs from L1/L2 to L5/S1 of each participant were obtained in the standing position during the medical examination. Radiographic features related to lumbar spondylosis, including OP, DHN, VS, and SL, were separately graded and classified into three groups: grade 0, normal; grade 1, mild change; and grade 2, severe change (Fig 1). The aggregate grades of each radiographic feature (OP, DHN, VS and SL) of the whole lumbar spine (from L1/L2 to L5/S1) was defined as ‘wOP’, ‘wDHN’, ‘wVS’ and ‘wSL’.

The sum of the grades of each radiographic feature at each intervertebral level was designated as the intervertebral grade (IG) to show the extent of LS at each intervertebral level. The aggregate grades of IG from L1/L2 to L5/S1 were defined as ‘wIG’ to show the extent of radiographic features of the entire lumbar spine. Lateral lumbar radiographs were also evaluated using the KL classification according to previous reports [4, 5], which defined radiographic change as KL 0, normal; KL 1, minimal OP only; KL 2, definite OP with some sclerosis of the anterior part of the vertebral plate; KL 3: marked OP and VS with slight DHN; and KL 4: large OP, marked VS, and marked DHN. The sum of KL grades of the whole lumbar spine (from L1/L2 to L5/S1) was defined as ‘wKL’.

All radiographs were evaluated by a board-certified orthopedic surgeon (JY) who was blinded to the participants’ information. Inter-observer reliability between two board-certified orthopedic surgeons (JY and NT) was examined using radiographs of 30 randomly selected participants as well as intra-observer reliability examination.

### Statistical analysis

Data were expressed as the mean ± standard deviation (SD). Shapiro-Wilk test was performed to test the data of normality of distribution. According to the Shapiro-Wilk test, age was analyzed using an independent sample t-test. The Mann-Whitney U test was used for body mass index (BMI), BMD, radiographic grade and health-related QOL scales. The chi-square test was performed to evaluate the distribution of the grades of each radiographic parameter. The post hoc test was performed to assess the probability values for each combination of independent category levels by using a Bonferroni correction to control for type I error inflation [16]. Logistic regression analysis was performed to identify the factors associated with the prevalence of LBP. Correlations between the novel elemental grading system and KL classification were analyzed using Spearman’s correlation coefficient. Statistical significance was set at P < 0.05. All statistical analyses were performed using IBM SPSS Statistics software (IBM Japan, Tokyo).

## Results

### Participant characteristics

A total of 300 inhabitants (79 men and 221 women; mean age: 72.0 years old) who underwent a medical examination in 2019 were enrolled in this study. 40 Participants with acute low back pain, lumbar scoliotic change, lumbar vertebral fracture of grade 2 or 3 according to semi-quantitative evaluation [17] and implants for lumbar spinal fusion surgery and any those who had missing data from the reported outcomes, were excluded from this study. A total of 260 (75 males, 185 females, mean age 71.5 ± 8.7 years old) participants were included in this study. The background data for the LBP- and LPB+ groups are shown in Table 1. There were no significant differences in age, sex, BMI, and BMD between the two groups. NRS and ODI were significantly higher in the LBP+ group than in the LBP group (P< 0.01, respectively). EQ-5D and EQ-VAS scores in the LBP+ group were significantly lower than those in the LBP group (P< 0.01, respectively).

**Table 1:** Participants’ characteristics according to the presence of low back pain.

	Overall	LBP-	LBP+	P-value
Age (years)	71.5 ± 8.7	71.1 ± 0.6	72.8 ± 1.0	0.15
Women (%)	185 (71.2%)	135 (71.4%)	50 (70.4%)	0.88
BMI (kg/m^2^)	22.8 ± 3.3	22.9 ± 3.4	22.6 ± 3.1	0.49
BMD (g/cm^2^)	0.5 ± 0.1	0.6 ± 0.1	0.5 ± 0.1	0.39
NRS	1.8 ± 2.1	0.8 ± 1.2	4.2 ± 1.9	<0.01
ODI (%)	12.0 ± 12.0	8.7 ± 10.0	20.6 ± 12.7	<0.01
EQ-5D	0.9 ± 0.2	0.9 ± 0.2	0.8 ± 0.2	<0.01
EQ-VAS	73.8 ± 17.0	76.6 ± 16.1	66.1 ± 16.9	<0.01

Data are expressed as mean ± standard deviation. P-value (vs. LBP-) was analyzed using an independent sample t-test for age and the Mann-Whitney U test for BMI, BMD, radiographic grade and health-related QOL scales. Women (vs. men) was evaluated by Chi-square test. BMI, body mass index; BMD, bone mineral density; NRS, numerical rating scale; LBP, low back pain; ODI, Oswestry Disability Index; EQ-5D, EuroQoL 5-Dimension; EQ-VAS, EuroQOL Visual Analogue Scale.

### Inter-, intra-observer reliability of novel elemental grading system

The inter-observer (JY-NT) and intra-observer reliability (JY-JY) and reproducibility coefficients of OP, DHN, VS, SL, IG, and KL were more than 0.7, indicating a strong correlation, respectively (Table 2).

**Table 2:** Inter-, intra-observer reliability of novel elemental grading system.

	OP	DHN	VS	SL	IG	KL
Inter-observer	0.88	0.83	0.88	0.83	0.75	0.77
Intra-observer	0.82	0.90	0.86	0.92	0.74	0.81

The inter-and intra-observer reproducibility in each radiographic feature was shown by the kappa coefficient. OP: osteophyte, DHN: disc height narrowing, VS: vertebral sclerosis, SL: spondylolisthesis, IG: intervertebral grade, KL: Kellgren Lawrence

### Radiographic features of lumbar spondylosis by novel elemental grading system

**Fig 2:**
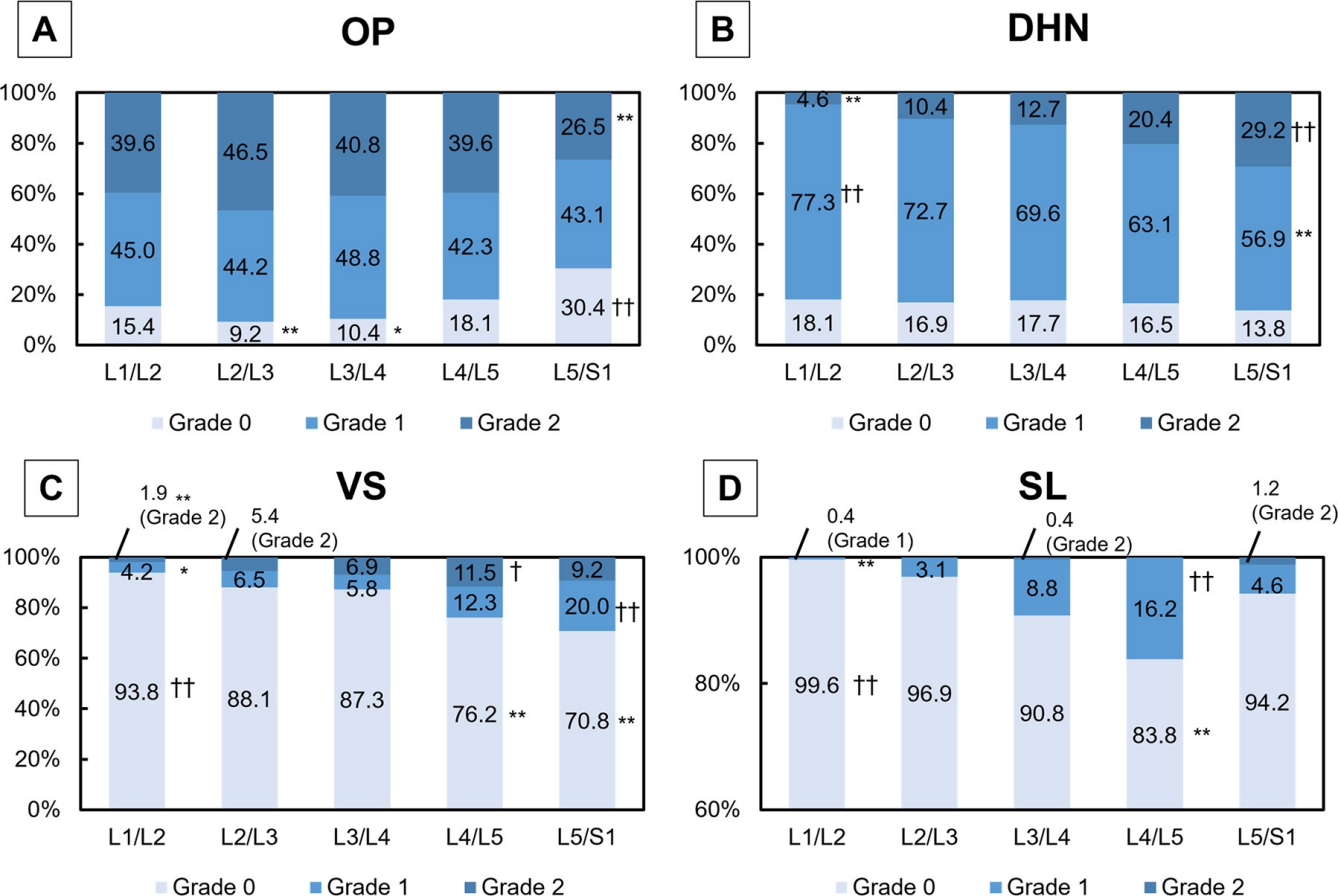
Distribution of radiographic features of lumbar spondylosis. Data are expressed by the percentage of intervertebral levels. A: osteophyte (OP), B: disc height narrowing (DHN), C: vertebral sclerosis (VS), D: spondylolisthesis (SL). Chi-square test showed significant differences among intervertebral level in all radiographic features (P<0.01). * indicates significantly lower than expected (*P<0.05, **P<0.01) and † indicates significantly higher than expected (†P<0.05, ††P<0.01) (by Bonferroni correction).

Significant differences in all radiographic parameters among the intervertebral levels were found (P<0.01, Fig 2). In the OP group, there was a significantly lower distribution of grade 0 than expected at L2/L3 (9.2%, P<0.01) and L3/L4 (10.4%, P<0.05), and a significantly higher distribution than expected at L5/S1 (30.4%, P<0.01) (Fig 2A). Grade 2 OP at L5/S1 was significantly less frequent than expected (26.5%, P<0.01). The DHN grade was predominantly grade 1 at all intervertebral levels (Fig 2B). There was a significantly lower distribution of grade 1 than expected at L5/S1 (56.9%, P<0.01), and a higher distribution than expected at L1/L2 (77.3%, P<0.01). The distribution of grade 2 DHN increased at lower levels and was significantly lower than expected at L1/L2 (4.6%, P<0.01) and higher than expected at the L5/S1 level (29.2%, P<0.01). VS was predominantly found at the lower lumbar level (Fig 2C). Grade 0 VS was significantly more frequent than expected at L1/L2 (93.9%, P<0.01) and less frequent than expected at L4/L5 (76.2%) and L5/S1 (70.8%) (P<0.01, respectively). The distribution of grade 1 VS was significantly lower than expected at L1/L2 (4.2%) (P<0.05) and higher than expected at L5/S1 (20.0%, P<0.01). A significantly lower distribution of grade 2 was seen at L1/L2 (1.9%, P<0.01), while a higher distribution of grade 2 was found at L4/L5 (11.5%, P<0.05). A significantly higher distribution of grade 0 SL than expected at L1/2 (99.6%) (P<0.01) and more distribution of grade 1 SL than expected at L4/5 (16.2%, P<0.01) were found (Fig 2D).

### Comparison of radiographic features between the LBP- and LBP+ groups

**Fig 3:**
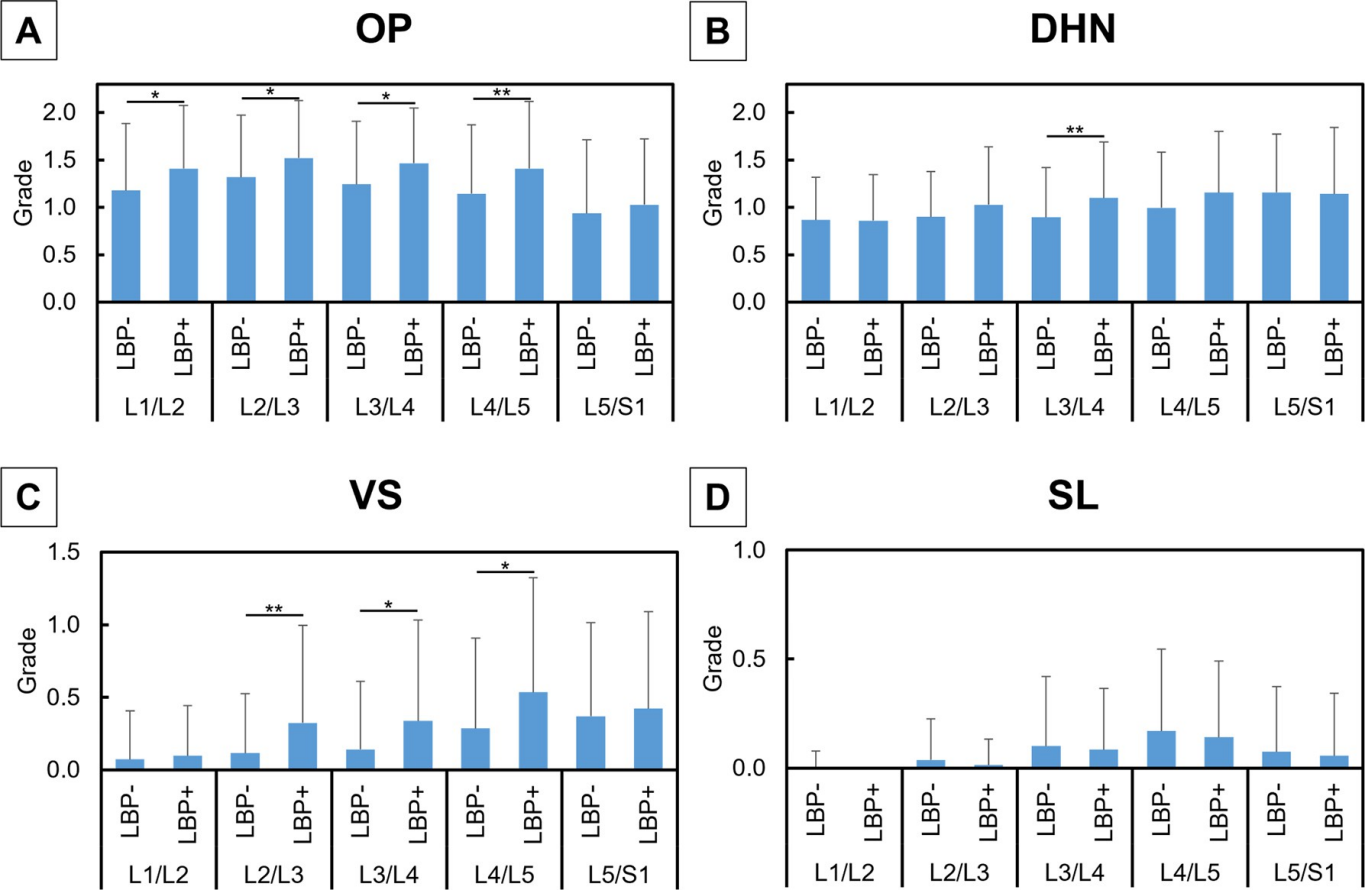
Grades of radiographic features with or without low back pain. Mean grades in the osteophytes (OP) (**A**), disc height narrowing (DHN) (**B**), vertebral sclerosis (VS) (**C**), and spondylolisthesis (SL) (**D**) with low back pain (LBP+) or without LBP (LBP-) at each intervertebral level are shown. Grades in each radiographic feature between the two groups were compared using the Mann-Whitney test. Data are expressed as mean ± standard deviation. *P<0.05 and **P<0.01.

**Fig 4:**
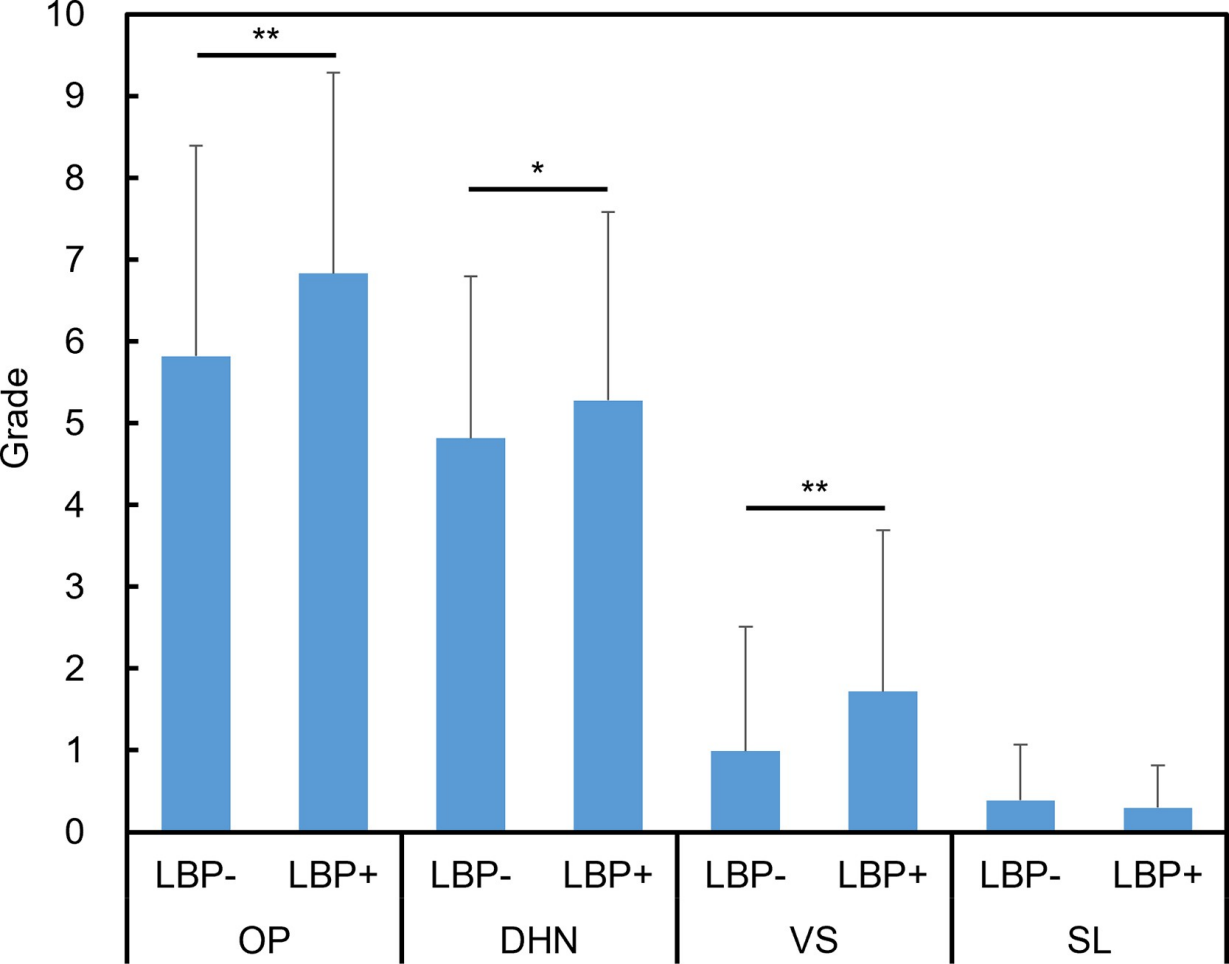
Radiographic features of whole lumbar with or without low back pain. Mean wOP (sum of grades of osteophytes from L1/L2 to L5/S1) (**A**), wDHN (sum of grades of disc height narrowing from L1/L2 to L5/S1) (**B**), wVS (sum of grades of vertebral sclerosis from L1/L2 to L5/S1) (**C**), and wSL (sum of grades of spondylolisthesis from L1/L2 to L5/S1) (**D**) with low back pain (LBP+) or without LBP (LBP-) at each intervertebral level are shown. Grades in each radiographic feature between the two groups were compared using the Mann-Whitney test. Data are expressed as mean ± standard deviation. *P<0.05 and **P<0.01.

The OP grade showed significant differences between the LBP- and LBP + groups at L1/L2, L2/L3, L3/L4 (P<0.05, respectively) and L4/L5 (P<0.01) (Fig 3A). A significant difference in the DHN grade was found only at L3/4 (P<0.01) (Fig 3B). VS grade also showed significant differences between the two groups at L2/L3 (P<0.01), L3/L4, and L4/L5 (P<0.05, respectively) (Fig 3C). No significant differences in SL grade were observed at any level (Fig 3D). In the whole lumbar analysis, the wOP (P<0.01), wDHN (P<0.05), and wVS (P<0.01) were significantly higher in the LBP+ group than in the LBP- group, while wSL grade showed no significant differences (Fig 4). Logistic regression analysis demonstrated that wVS was a significant independent factor associated with LBP (Table 3). Between VS and the other parameters, there were significant differences on their distribution analyzed by chi-square test (P<0.01). In the grade 2 VS, most cases were accompanied by grade 2 OP (86.8%) and grade 2 DHN (97.8%) with significantly higher frequency than expected (P<0.01).

**Table 3:** Logistic regression analysis for the factors associated with low back pain.

	P-value	Odds ratio	95% Confidence interval	
			Lower	Upper
wVS	0.003	1.263	1.082	1.474

wVS (sum of grades of vertebral sclerosis from L1/L2 to L5/S1)

### Comparison between novel elemental grading system and KL between the LBP- and LBP+ group

There were strong correlations between IG evaluated by the novel elemental grading system and KL grades, especially at the lower lumbar level (r = 0.70–0.84, P<0.01, Table 4). The wIG and wKL also showed a strong correlation (r = 0.85, P<0.01).

**Table 4:** Correlation between novel elemental grading system and KL classification for evaluation of radiographic lumbar spondylosis.

	L1/L2	L2/L3	L3/L4	L4/L5	L5/S1	Whole lumbar
r	0.70	0.76	0.78	0.83	0.84	0.85
P-value	<0.01	<0.01	<0.01	<0.01	<0.01	<0.01

The correlation between the intervertebral grade (IG) graded by the novel elemental grading system and Kellgren Lawrence grading (KL) was analyzed using Spearman’s correlation coefficients (r).

**Fig 5:**
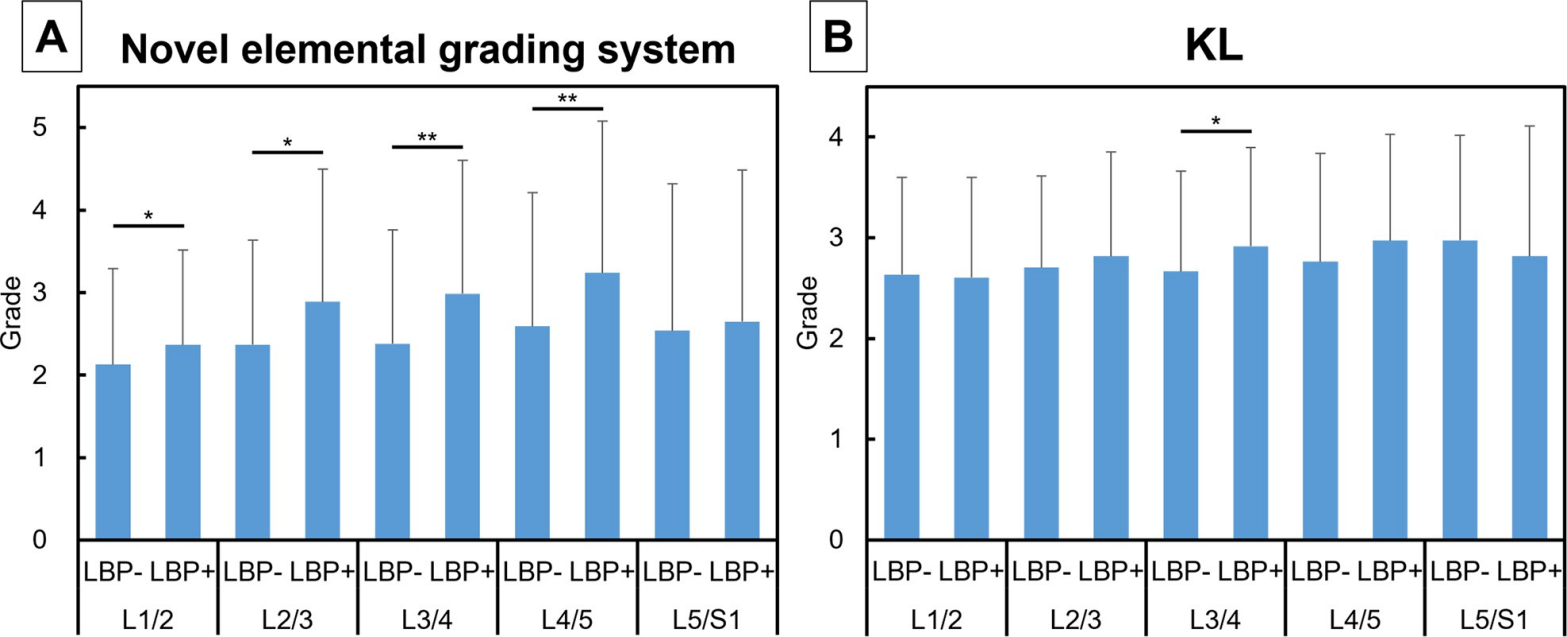
Intervertebral grade and Kellgren Lawrence grade with or without low back pain. Mean intervertebral grade (IG) in the elemental grading system (**A**) and Kellgren Lawrence (KL) grade (**B**) with low back pain (LBP+) or without LBP (LBP-) at each intervertebral level are shown. Data are expressed as mean ± standard deviation. Differences in IG or KL grade at each disc level were statistically evaluated using the Mann-Whitney test. *P<0.05 and **P<0.01.

**Fig 6:**
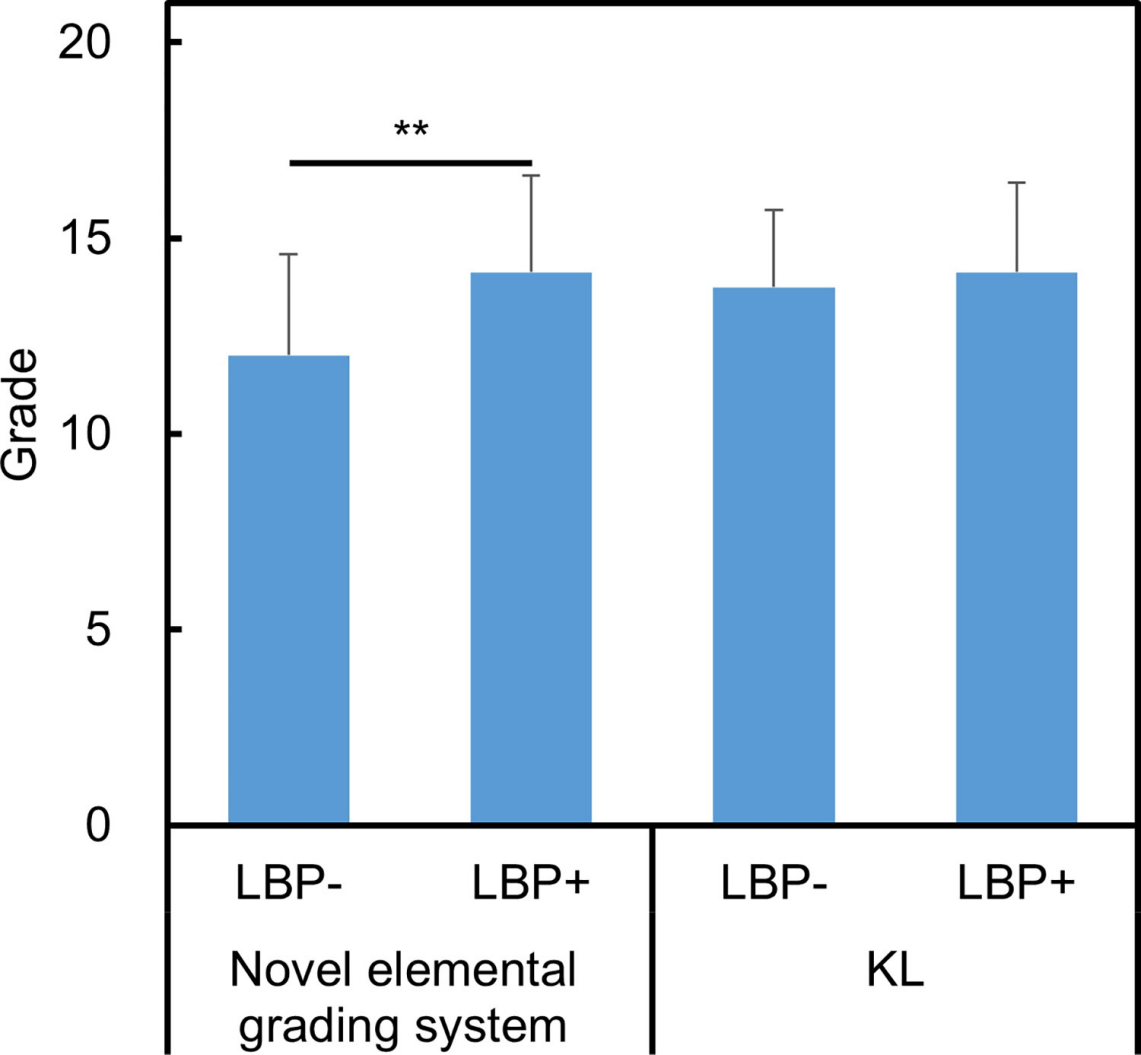
Intervertebral grade and Kellgren Lawrence grade of whole lumbar spine with or without low back pain. Mean intervertebral grade of whole spine (wIG) in the novel elemental grading system (**A**) and Kellgren Lawrence grade of whole spine (wKL) (**B**) with LBP (LBP+) or without LBP (LBP-) was shown. Data are expressed by mean ± standard deviation. Differences between the groups were statistically evaluated using Mann-Whitney test. *P<0.05 and **P<0.01.

The LBP+ group showed a significantly higher IG at L1/L2 and L2/L3 (P<0.05, respectively) and L3/L4, L4/L5 (P<0.01, respectively) than the LBP- group (Fig 5A), whereas a significant difference in KL grade between the two groups was only found at the L3/4 level (P<0.05) (Fig 5B). wIG was significantly higher in the LBP+ group than in the LBP- group (P<0.01), while no significant difference was found in wKL (Fig 6).

### Correlation between novel elemental grading system and subjects reported outcomes

There was a significant, but weak (r = 0.1–0.3) correlation between radiographic features and NRS, ODI, and EQ-5D scores (Table 5). However, no significant correlation was found with the EQ-VAS score.

**Table 5:** Correlation between radiographic features of lumbar spondylosis and NRS or health-related QOL.

	Novel elemental grading system					KL
	OP	DHN	VS	SL	IG	
NRS	0.17**	0.12*	0.18**	0.02	0.18**	0.12*
ODI	0.19**	0.15*	0.11	0.11	0.20**	0.15*
EQ-5D	-0.14*	-0.21**	-0.11	-0.086	-0.19**	-0.22**
EQ-VAS	-0.076	-0.074	-0.025	-0.12	-0.086	-0.089

NRS: numerical rating scale; ODI: Oswestry Disability Index; EQ-5D: EuroQOL 5-Dimension; EQ-VAS: EuroQOL Visual Analogue Scale; OP: Osteophyte; DHN: Disc Height Narrowing; VS: Vertebral Sclerosis SL: Spondylolisthesis, IG: Intervertebral Grade, KL: Kellgren Lawrence.

*P<0.05

**P<0.01

## Discussion

In this study, a novel elemental grading system was used to evaluate radiographic lumbar spondylosis among inhabitants of a typical mountain village. This study showed that the radiographic grades of OP, DHN, and VS in the LBP+ group were significantly higher than those in the LBP- group, while SL grade did not show a significant difference. The wIG of the LBP+ group was significantly higher than that of the LBP- group, while wKL did not reach significance. The novel elemental grading system might have a greater association with LBP by individually evaluating each radiographic feature.

However, the association between OP and LBP remains controversial [3, 18]. Lee et al. [19] reported that there was no significant association between OP and LBP among the middle-aged population or male subjects, while the presence of OP was associated with LBP only in elderly female subjects over 70 years, which is the majority population of this study. In accordance with the results of Lee’s study, this study showed that OP grades from L1/L2 to L4/L5 and wOP in the LBP+ group were significantly higher than those in the LBP- group.

Intervertebral disc height narrowing has been reported to increase constantly with age [20]. Goode et al. reported that the prevalence of DHN at a mild or greater severity was more than 73.3% in subjects aged 65 years or older [21]. Accumulating evidence shows a significant association between DHN and LBP [8, 18, 20, 22, 23]. A systemic review by Raastad et al. also concluded that DHN was significantly associated with LBP [3]. In accordance with previous studies, our study showed that the DHN grade at L3/4 and wDHN in the LBP+ group was significantly higher than that in the LBP- group.

Furthermore, a previous study reported that the association between VS and LBP also remains controversial [3, 20]. Inaoka et al. [23] reported a significantly higher incidence of VS in patients with LBP than in those without LBP. Our study also showed that the presence of VS was the predominant factor associated with LBP among the radiographic features of LS by logistic regression analysis. In this study, most cases of grade 2 VS were accompanied by grade 2 OP and grade 2 DHN. Therefore, the most severe VS in this study reflected severe spondylosis, which is reported to have significant association with LBP [3, 5].

In a systematic review, Raastad et al. reported a significant association between SL and LBP in an occupation-based study, but a weak association in a community-based study [3]. He et al. reported that SL was significantly associated with low back pain in men, whereas this association was not statistically significant in women in a population-based study of the elderly [24]. These previous studies suggest that SL may be a cause of LBP in a limited population. Our population-based study, in which the prevalence of SL was limited (28.5%) and 71.2% of participants were women, showed that there was no significant difference in SL grade between the LBP- and LBP+ groups.

Additionally, in our study, IG, which is the sum of the grades of OP, DHN, VS, and SL was used to evaluate the extent of LS at each intervertebral level. IG from L1/L2 to L4/L5 in the LBP+ group was significantly higher than that in the LBP- group, whereas KL showed a significant difference only at L3/4. Raastad et al. reported that lumbar spondylosis was significantly associated with LBP among community-based populations [3]. Previous reports also concluded that a severe KL grade may contribute to lower back pain [6, 25]. In this study, the mean grade of wIG in the LBP+ group was significantly higher than that in the LBP- group, while wKL did not reach a significant difference. This study showed that KL grade 3 accounted for the majority (67.7% of total) of disc levels as the disc level with mild DHN was classified as KL grade 3. Alternately, the IG of our system varies depending on the extent of each radiographic feature, even with mild DHN. Therefore, the novel elemental grading system may be more precise in reflecting the presence of LBP than the KL system by individually evaluating each radiographic feature of LS.

This study has several limitations. First, this study was conducted in a mountain village, where many inhabitants are typically engaged in forestry. Therefore, the occupation ratio would differ as compared with that of the general Japanese population. Second, the majority of the subjects were elderly women. Therefore, gender differences might have influence on the results of this study.

## Conclusion

The novel elemental grading system of LS that individually grades each radiographic feature would more accurately reflect the participant’s LBP than the KL classification. This simple grading system may contribute to future epidemiological studies to evaluate the extent of LS on lumbar radiographs.

## Supporting information

S1 File 11-grade pain intensity numerical rating scale (NRS). doi:10.1097/01.brs.0000164099.92112.29.
(DOCX)

S2 File EuroQOL 5-Dimension (EQ-5D) and EuroQOL Visual Analogue Scale (EQ-VAS). https://doi.org/10.1016/0168-8510(90)90421-9; doi:10.1093/bmb/ldq033.
(DOCX)

S3 File Oswestry Disability Index (ODI). doi:10.1097/00007632-200011150-00017.
(DOCX)

S4 File Study data. (XLSX)
