# Supplementary material for: Novel elemental grading system for radiographic lumbar spondylosis in a population based-cohort study of a Japanese mountain village
Source: PLoS One. 2022 Jun 28;17(6):e0270282. doi: 10.1371/journal.pone.0270282 (PMC9239436; doi:10.1371/journal.pone.0270282)
Supplement: S1 File — doi:10.1097/01.brs.0000164099.92112.29. (DOCX) [file pone.0270282.s001.docx]

Q1.

Do you have low back pain lasting more than three months?

Yes No

Q2.

On a scale of 0 to 10, with 0 being no pain at all and 10 being the worst possible pain, how would you rate your low back pain?


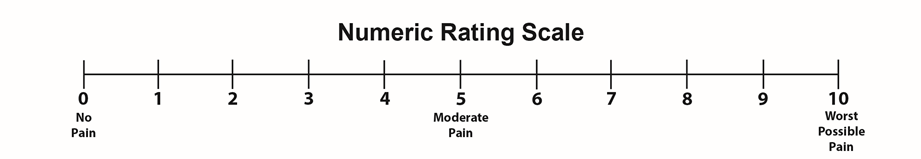


Farrar JT, Young JP, Jr., LaMoreaux L, Werth JL, Poole RM. Clinical importance of changes in chronic pain intensity measured on an 11-point numerical pain rating scale. Pain. 2001;94(2):149-58. Epub 2001/11/03. doi: 10.1016/s0304-3959(01)00349-9. PubMed PMID: 11690728.
