# Supplementary material for: Novel elemental grading system for radiographic lumbar spondylosis in a population based-cohort study of a Japanese mountain village
Source: PLoS One. 2022 Jun 28;17(6):e0270282. doi: 10.1371/journal.pone.0270282 (PMC9239436; doi:10.1371/journal.pone.0270282)
Supplement: S3 File — doi:10.1097/00007632-200011150-00017. (DOCX) [file pone.0270282.s003.docx]

**Please answer every section. Select one number only in each section that most closely describes you today.**

**Section 1 – Pain intensity**

1. I have no pain at the moment.
2. The pain is very mild at the moment.
3. The pain is moderate at the moment.
4. The pain is fairly severe at the moment.
5. The pain is very severe at the moment.
6. The pain is the worst imaginable at the moment.

**Section 2 – Personal care (washing, dressing, etc.)**

1. I can look after myself normally without causing extra pain.
2. I can look after myself normally but it causes extra pain.
3. It is painful to look after myself and I am slow and careful.
4. I need some help but manage most of my personal care.
5. I need help every day in most aspects of self-care.
6. I do not get dressed, I wash with difficulty and stay in bed.

**Section 3 – Lifting**

1. I can lift heavy weights without extra pain.
2. I can lift heavy weights but it gives extra pain.
3. Pain prevents me from lifting heavy weights off the floor but I can manage if they are conveniently positioned, e.g. on a table.
4. Pain prevents me from lifting heavy weights but I can manage light to medium weights if they are conveniently positioned.
5. I can lift very light weights.
6. I cannot lift or carry anything at all.

**Section 4 – Walking**

1. Pain does not prevent me walking any distance.
2. Pain prevents me from walking more than 1 mile.
3. Pain prevents me from walking more than 1/2 mile.
4. Pain prevents me from walking more than 100 yards.
5. I can only walk using a stick or crutches.
6. I am in bed most of the time.

**Section 5 – Sitting**

1. I can sit in any chair as long as I like.
2. I can only sit in my favourite chair as long as I like.
3. Pain prevents me sitting more than one hour.
4. Pain prevents me from sitting more than 30 minutes.
5. Pain prevents me from sitting more than 10 minutes.
6. Pain prevents me from sitting at all.

**Section 6 – Standing**

1. I can stand as long as I want without extra pain.
2. I can stand as long as I want but it gives me extra pain.
3. Pain prevents me from standing for more than 1 hour.
4. Pain prevents me from standing for more than 30 minutes.
5. Pain prevents me from standing for more than 10 minutes.
6. Pain prevents me from standing at all.

**Section 7 – Sleeping**

1. My sleep is never disturbed by pain.
2. My sleep is occasionally disturbed by pain.
3. Because of pain I have less than 6 hours sleep.
4. Because of pain I have less than 4 hours sleep.
5. Because of pain I have less than 2 hours sleep.
6. Pain prevents me from sleeping at all.

**Section 8 – Sex life (if applicable)**

1. My sex life is normal and causes no extra pain.
2. My sex life is normal but causes some extra pain.
3. My sex life is nearly normal but is very painful.
4. My sex life is severely restricted by pain.
5. My sex life is nearly absent because of pain.
6. Pain prevents any sex life at all.

**Section 9 – Social life**

1. My social life is normal and gives me no extra pain.
2. My social life is normal but increases the degree of pain.
3. Pain has no significant effect on my social life apart from limiting my more energetic interests, e.g. sport, etc.
4. Pain has restricted my social life and I do not go out as often.
5. Pain has restricted my social life to my home.
6. I have no social life because of pain.

**Section 10 – Travelling**

1. I can travel anywhere without pain.
2. I can travel anywhere but it gives me extra pain.
3. Pain is bad but I manage journeys over two hours.
4. Pain restricts me to journeys of less than one hour.
5. Pain restricts me to short necessary journeys under 30 minutes.
6. Pain prevents me from travelling except to receive treatment.

**Reference**

Fairbank JCT, Pynsent PB. The Oswestry Disability Index. Spine. 2000;25(22).
